# Supplementary material for: Full-range birefringence control with piezoelectric MEMS-based metasurfaces
Source: Nat Commun. 2022 Apr 19;13:2071. doi: 10.1038/s41467-022-29798-0 (PMC9018774; doi:10.1038/s41467-022-29798-0)
Supplement: Supplementary file 1 — Supplementary Information [file 41467_2022_29798_MOESM1_ESM.pdf]

Supplementary Information

Full-range birefringence control with piezoelectric MEMS-based metasurfaces

Chao Meng, [1†] Paul C. V. Thrane, [1,2†] Fei Ding,[1*] and Sergey I. Bozhevolnyi[1*]

[1]*Centre for Nano Optics, University of Southern Denmark, Campusvej 55, Odense DK-5230, Denmark*

[2]*SINTEF Microsystems and Nanotechnology, Gaustadalleen 23C, 0737 Oslo, Norway*

[†]These authors contributed equally to this work

[*]Corresponding author emails: feid@mci.sdu.dk (F.D.); seib@mci.sdu.dk (S.I.B.)

The PDF file includes:

Supplementary Table 1

S1. Optimization of the nanobrick dimensions

S2. MEMS-OMS assembly and pre-characterizations

S3. Air gap estimation

S4. Experimental setup

S5. Polarization Conversion for fixed incident LP state |*y*> (θ_{LP} = 90°) and DWP orientation θ_{DWP} = 45°.

S6. Higher quality of four typical polarization states (i.e., |*x*>, |*y*>, |*r*>, |*l*>) with DWP orientation θ_{DWP} =46°, for fixed incident LP state |*y*> (θ_{LP} = 90°).

S7. Versatile polarization transformations

References

Other Supplementary Material for this manuscript includes the following:

Supplementary Videos S1 to S6

Supplementary Table 1

Supplementary Table 1: Overview of key performance characteristics estimated for some demonstrated devices with tunable birefringence.

Principle	*Stimulus*	*Birefringence range*	*Efficiency*	*Operation λ, Bandwidth*	*Response time*	*Ref.*
Plasmonic OMS and tunable FP cavity, in reflection	Electrical	2π	~75 %	800 nm, BW ~100 nm	< 400 µs	This work
Plasmonic PB metasurface with liquid crystals, in reflection	Electrical	π	~40 %	633 nm, BW ~110 nm	100 ms	1
Tri-layer black phosphorus in a FP cavity, in reflection	Electrical	Half Poincaré sphere	< 10 %	1440 nm, BW < 10 nm	—	2
Transient Nonlinear anisotropic effect in gold nanorods metamaterial, in transmission	Optical	π	< 20 %	700 nm, BW ~10 nm	10 ps	3
Transient symmetry breaking in gold metasurface, in transmission	Optical	< 1°	~2 %*	650 nm, BW 100 nm	<1 ps	4
Transient anisotropic effect in ITO-integrated gold nanostructures, in transmission	Optical	π / 7	—	1.23 µm, BW ~25 nm	600 fs	5
Chiral metasurface on vibrating silicon membrane with FP cavity underneath, in reflection	Electrical	π / 60	~1 %*	1.45 µm, BW ~5 nm	3 µs	6

* The efficiency listed for Ref. 4 and 6 is derived from the normalized reflectance or transmittance change in one specific polarization channel, to indicate their polarization conversion capabilities.

S1. Optimization of the nanobrick dimensions

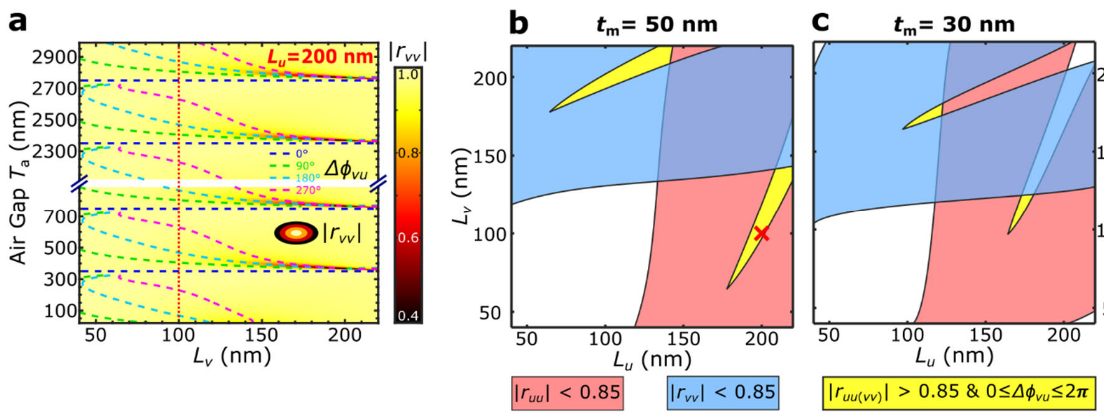

Fig. S1. Choice of nanobrick dimensions. a Reflection amplitude $|r_{vv}|$ and birefringence ($\Delta\phi_{vu}$) as a function of the nanobrick side length L_v and air gap T_a for the other side length L_u = 200 nm. Color map is related to the reflection amplitude, while colored dashed lines represent constant birefringence contours, similarly to Fig. 1b. The vertical, dotted line corresponds to the selected nanobrick dimensions $L_u \times L_v$ = 200 nm × 100 nm. **b-c** Optimization of the nanobrick thickness. The L_u, L_v parameter space is divided into several regions, for nanobrick thicknesses of t_m = 50 nm (**b**) and 30 nm (**c**). The red and blue areas indicate respectively where $|r_{uu}|$ < 0.85 and $|r_{vv}|$ < 0.85 for any value of T_a. The yellow areas indicate where the reflection amplitudes are large for all polarization states (i.e., $|r_{uu(vv)}|$ > 0.85) and where additionally the birefringence $\Delta\phi_{vu}$ can be adjusted fully between 0 and 2π by varying the air gap T_a. Note that in these yellow areas, one polarization is almost entirely reflected by the OMS while the orthogonal polarization is partly transmitted, as can be seen from Fig. S2c. The red cross in (**b**) indicates the selected dimension L_u = 200 nm and L_v = 100 nm.

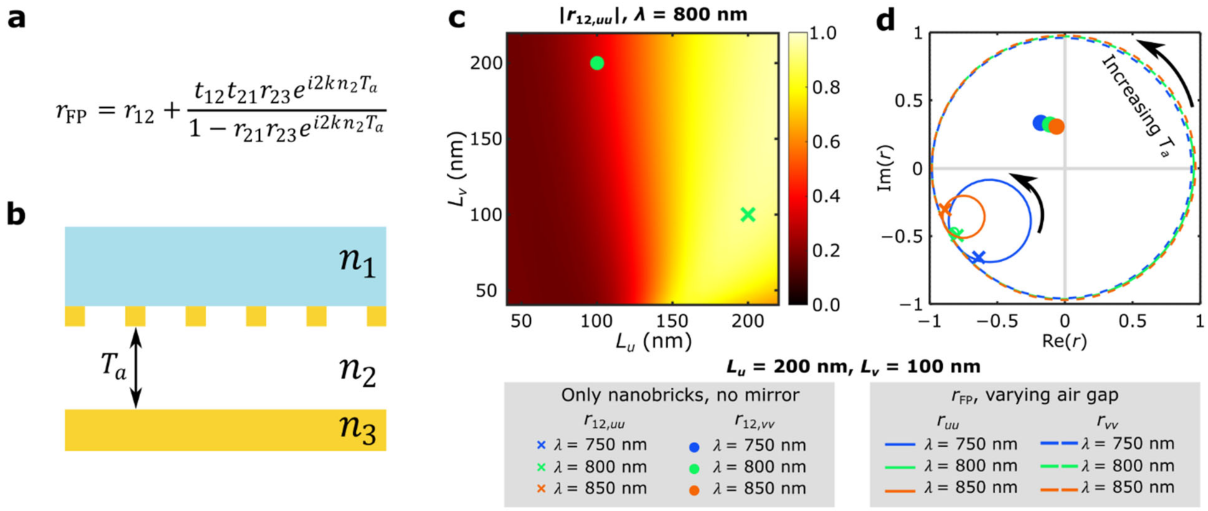

Fig. S2. DWP operating in the FP regime and corresponding tunable reflection coefficients. a FP equation of the total reflection coefficient for light normally incident on a multilayer structure consisting of

medium 1, OMS layer, media 2 and 3 as illustrated in (**b**). Here, r_{FP} is the complex reflection coefficient of the hybrid MEMS-OMS structure, and r_{mn}/t_{mn} represent the respective complex reflection/transmission coefficients for the interface between media m and n with light incident from medium m. The OMS layer is located between glass/air interface, and media 1, 2 and 3 are respective glass, air, and gold materials, thus the interface 12 represent a complex glass/OMS/air interface, and interface 23 is bare air/gold interface. **b** Side view of the schematic DWP consisting of a glass substrate (medium 1), an OMS layer (gold nanobrick array), an air gap T_a (medium 2), and MEMS mirror coated with thick gold film (medium 3). **c** Reflection amplitude $|r_{12}|$ (related to the interface of medium 1/OMS/medium 2) for input linearly $|u\rangle$ polarized light as a function of the nanobrick dimensions (L_u, L_v) at λ = 800 nm. **d** Total reflection coefficient r_{FP} in the complex plane with L_u = 200 nm, L_v = 100 nm, t_m = 50 nm, and Λ = 250 nm for three different wavelengths of λ = 750, 800 and 850 nm. Filled crosses and circles in (**c**, **d**) show r_{12} for incident light linearly polarized along respective u and v directions (i.e., along either nanobrick sides), while solid and dashed lines represent the variation of r_{FP} as a function of the air gap T_a, for incident light linearly polarized along u and v directions, respectively. Arrows indicate the direction r_{FP} traverses along the trajectory as T_a increases.

S2. MEMS-OMS assembly and pre-characterizations

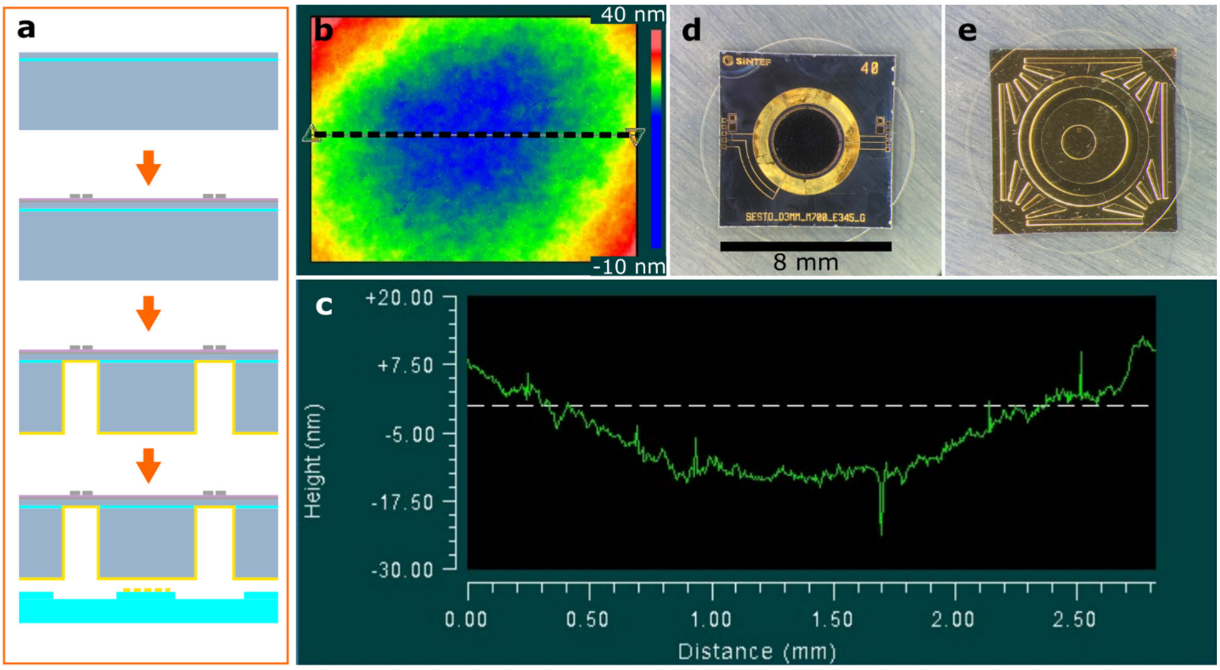

Fig. S3. MEMS-OMS fabrication and MEMS mirror characterization. **a** Simplified cross-section illustration of the MEMS-OMS fabrication process: preparation of a silicon-on-insulator wafer; a bottom electrode layer, PZT layer and top electrodes are deposited on the device layer; subsequently an annular trench is etched through the substrate and oxide layers, before the wafer is covered in a reflective gold coating – resulting in a central mirror suspended by an annular membrane; finally the wafer is diced into individual chips that are glued to glass substrates with an OMS. The glass substrates have a 10 μm deep annular trench to reduce issues with big particle contaminants between the MEMS mirror and glass surface. **b-c** White light interferometric measurement (Zygo NewView 6300) of the MEMS mirror surface. (**c**) shows the profile along the dashed line in (**b**). **d-e** Optical microscope images of the MEMS chip on the glass substrate.

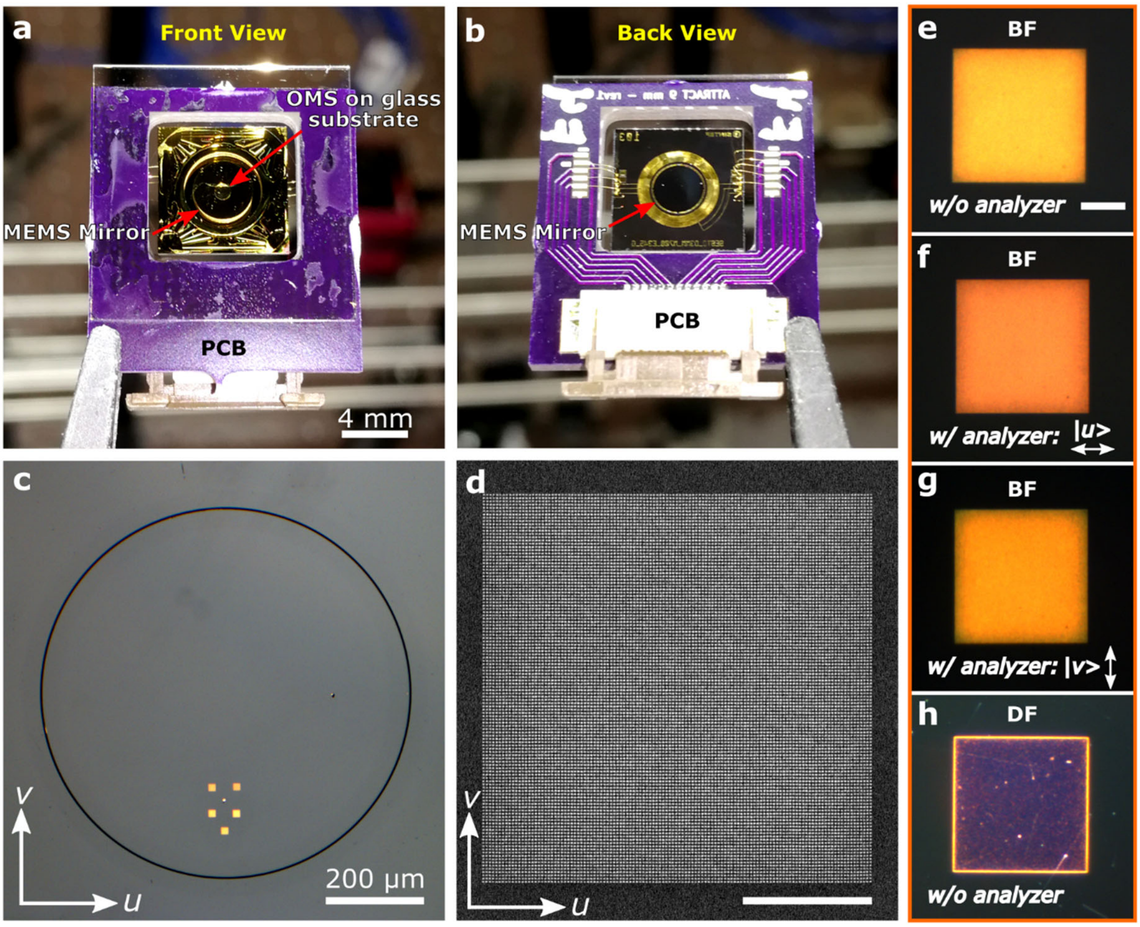

Fig. S4. MEMS-OMS-based DWP assembly. a-b Typical photos of the MEMS-OMS-based DWP assembly consisting of the OMS fabricated on a glass substrate, an ultra-flat piezoelectric MEMS mirror, and a printed circuit board (PCB) for electrical connection. **c-d** Optical microscopy (**c**) and SEM (**d**) images of the OMS representing the 30 μm by 30 μm and 250-nm-period array of same-sized gold nanobricks (L_u = 200 nm, L_v = 100 nm) designed for DWP, fabricated atop a 10-μm-high circular pedestal on the glass substrate, and used in the MEMS-OMS-based DWP assembly. Scalebar in (**d**) is 10 μm. **e-h** Bright and dark field optical reflection microscopy images of the OMS fabricated on the glass substrate before assembling to MEMS mirror, captured with (**f**, **g**) and without (**e**, **h**) polarization analyzer. Scale bar in (**e**) is 10 μm, which also applies to (**f-h**).

S3. Air gap estimation

For larger air gaps (i.e., $T_a > 100$ nm for 800-nm wavelength), the underlying physics dominate the optical response of the MEMS-OMS-based DWP slip into a FP regime in which the MEMS-OMS structure can be considered as a hybrid FP cavity consisted of multilayers including a thick gold layer, an air gap layer (with thickness T_a), an OMS layer, and a glass layer (Fig. S2b), thus the optical response of the MEMS-OMS structure is governed by the FP equation[7–9], as shown in Fig. S2a:

$$r_{\text{FP}} = r_{12} + \frac{t_{12}t_{21}r_{23}e^{i2\mathbf{k}n_2T_a}}{1-r_{21}r_{23}e^{i2\mathbf{k}n_2T_a}} \quad (1)$$

If we consider the OMS layer as a periodic structured infinitesimally layer, we have the following relationship[9–11]: $t_{12} = 1 + r_{12}$ and $t_{21} = 1 + r_{21}$, then equation (1) could be simplified as:

$$r_{\text{FP}} = r_{12} + \frac{(1 + r_{12})(1 + r_{21})r_{23}e^{i2\mathbf{k}n_2T_a}}{1 - r_{21}r_{23}e^{i2\mathbf{k}n_2T_a}} = \frac{r_{12} + (1 + r_{12} + r_{21})r_{23}e^{i2\mathbf{k}n_2T_a}}{1 - r_{21}r_{23}e^{i2\mathbf{k}n_2T_a}}$$

Assume $|r_{23}| = 1$, we could then always get $r_{23}e^{i2\mathbf{k}n_2T_a} = -1$, for a series of periodic-distributed air gaps. At these air gaps, the total reflection coefficient from the MEMS-OMS structure is:

$$r_{\text{FP}} = \frac{r_{12} + (1 + r_{12} + r_{21}) \times (-1)}{1 - r_{21} \times (-1)} = \frac{-(1 + r_{21})}{(1 + r_{21})} = -1$$

Finally, we get $r_{\text{FP}} = -1$, which implies, for a series of periodic-distributed air gaps, the MEMS-OMS structure operates as a featureless perfect mirror, regardless of the dimensions and arrangement of the OMS structures, as if the OMS layer is not present! Note that the prerequisite for arriving at a perfect mirror operation is $|r_{23}| = 1$, which is approximately true for the air/gold interface implemented in the current MEMS-OMS-based DWP design (Fig. S5). The reflection amplitude for the air/gold(ideal) interface is close to 1.0 (i.e., >0.98) within the investigated wavelength range of 650-1000 nm. One would also notice that even if the practical gold materials may have larger losses, the influences are still negligible (Fig. S5).

The mirror-like operation described above have almost no correlation with the nanobrick dimensions in the OMS layer and the practical gold material losses, however it is strongly influenced by the wavelength (Fig. S6) which could be clearly observed by the condition $r_{23}e^{i2\mathbf{k}n_2T_a} = -1$ in which $\mathbf{k}$ is the wavevector in vacuum and solely determined by the wavelength λ. From equation (1), we could also conclude that the response of the MEMS-OMS structures is periodic with respect to the air gap T_a.

The relationship between the air gap T_a and wavelength λ connected to the mirror-like operation provides a reliable approach for in situ estimating the practical air gap sizes in each measurement. For example, in experiment, if we incident a broadband light with LP $|y\rangle$ state onto the MEMS-OMS DWP, regardless of the DWP orientation, we could always expect some reflection minimum by detecting the reflected light with a polarization analyzer along $|x\rangle$ direction (i.e., orthogonal to input LP $|y\rangle$ state) at specific wavelengths. This information builds up a one-to-one mapping between the practical air gaps and the actuation voltages applied to the MEMS mirror in each measurement (Fig. S7).

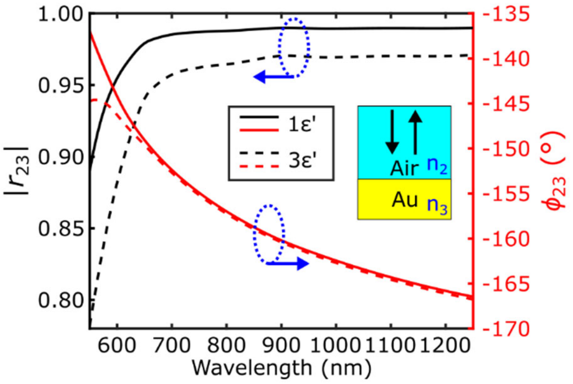

Fig. S5. Calculated reflection coefficients of the air/gold interface. Reflection amplitudes (black) and phases (red) as a function of the wavelength. Solid and dashed lines represent calculations with gold materials ideal (1ε') and damping rate increased (3ε', the imaginary part of the ideal gold permittivity is increased by a factor of three), respectively.

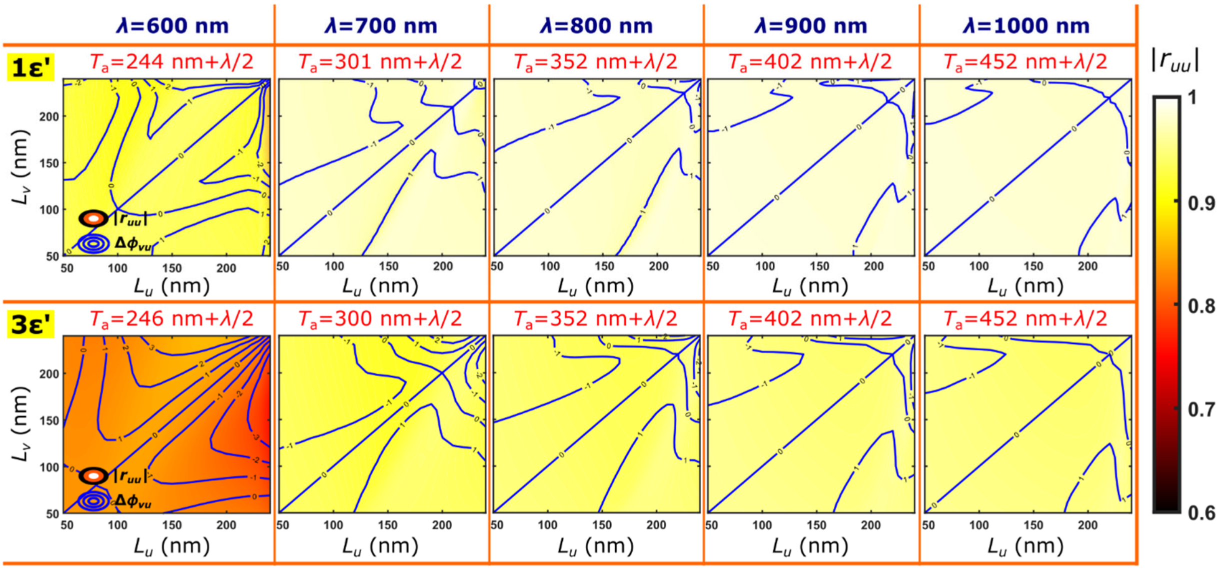

Fig. S6. Mirror-like operation of the MEMS-OMS structures at specific air gaps for different wavelengths. Calculated reflection amplitude $|r_{uu}|$ (color map) and birefringence $\Delta\phi_{vu}$ (blue contour lines) as a function of nanobrick dimensions (L_u, L_v) for Λ = 250 nm and t_m = 50 nm. The top and bottom panels represent simulations with gold material ideal (1ε') and damping rate increased (3ε', the imaginary part of the ideal gold permittivity is increased by a factor of three), respectively.

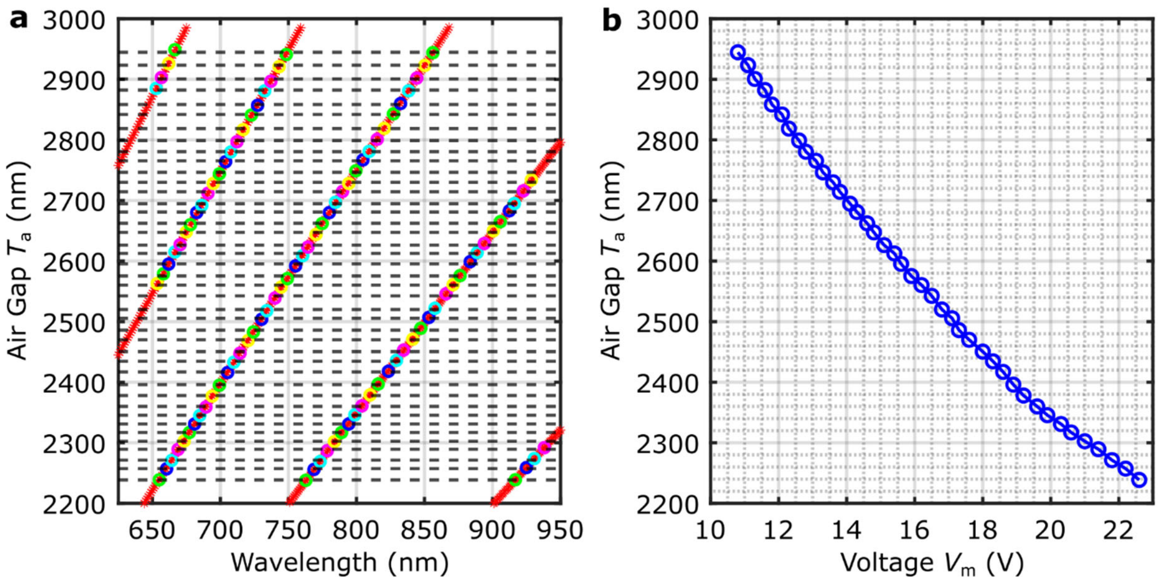

Fig. S7. Air gap estimation. a Estimating practical air gaps at different actuation voltages according to the mirror-like operations in the MEMS-OMS DWP. The red lines represent theoretical relationship between the air gap T_a and the wavelength for mirror operation, while the colored circles are extracted wavelengths exhibiting mirror-like operation obtained from each measurement (i.e., for different actuation voltages V_m). The dashed horizontal lines are the least square fit between the experimental and theoretical data for estimating the air gap sizes. **b** Estimated air gap size T_a as a function of the actuation voltage V_m.

S4. Experimental setup

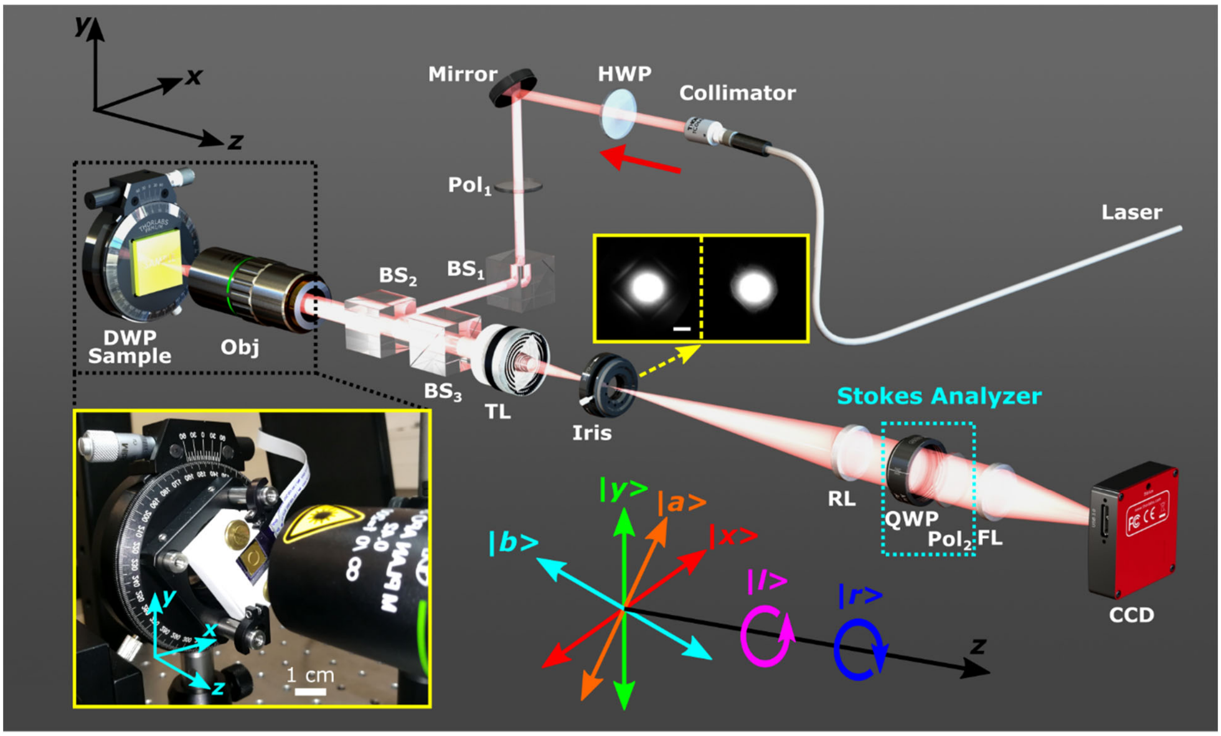

Fig. S8. Experimental setup for characterizing MEMS-OMS DWP. Laser: Fiber coupled laser sources; Collimator: fiber collimator (TC06APC-780, Thorlabs); HWP: Half-wave plate (AHWP10M-980, Thorlabs); Mirror: Protected silver mirror (PF10-03-P01, Thorlabs); $Pol_{1,2}$: Linear Polarizer (LPNIR050-MP2, Thorlabs); $BS_{1,2,3}$: Beam splitter (CCM1-BS014/M); Obj: Objective (M Plan Apo 20×/0.42, Mitutoyo); TL: Tube lens (TTL200-S8, Thorlabs); Iris: Spatial filter (SM1D12SZ, Thorlabs); RL: Relay lens (AC254-200-B-ML, f = 200 mm, Thorlabs); QWP: Quarter-wave plate (AQWP10M-980, Thorlabs); FL: Flip lens (AC254-100-B-ML, f = 100 mm, Thorlabs); CCD: CCD camera (DCC1545M, Thorlabs). The iris is located at the first conjugated image plane for filtering out the area of interest in the measurement, and the Stokes analyzer consisting of a QWP and a Pol_2 is implemented for full Stokes polarimetry. The lower-left inset is a typical optical image of the DWP sample mounted on a high precision rotation stage (PRM1, Thorlabs). The lower-right inset is the definition of the polarization bases used in the polarization characterization, i.e., (|x>, |y>), (|a>, |b>), and (|r>, |l>).

S5. Polarization Conversion for fixed incident LP state |*y*> (θ_{LP} = 90°) and DWP orientation θ_{DWP} = 45°.

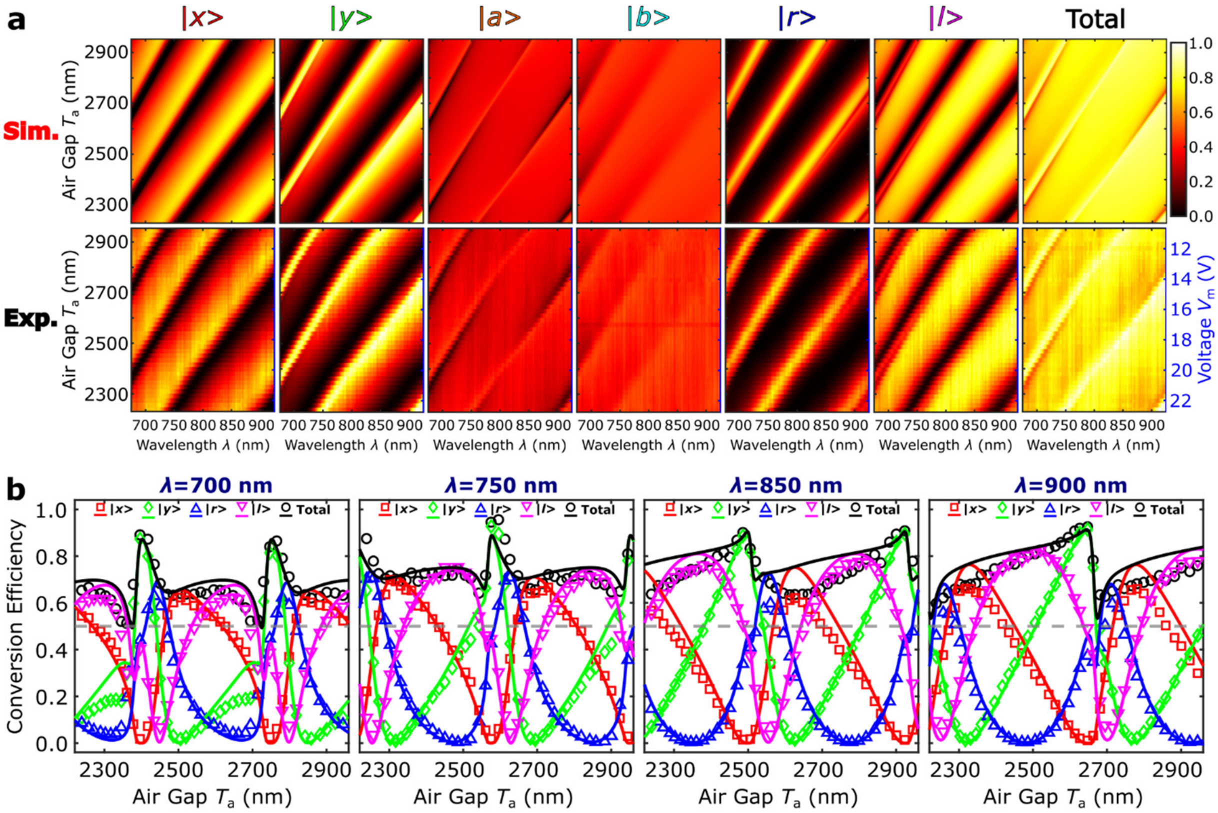

Fig. S9. Polarization conversion efficiencies for fixed input LP |*y*> state (θ_{LP} = 90°) and DWP orientation θ_{DWP} = 45°. a Calculated (above) and measured (below) polarization conversion efficiencies as a function of the air gap T_a (or actuation voltage V_m) and light wavelength λ. **b** Calculated (lines) and measured (markers) polarization conversion efficiencies as a function of T_a for the light wavelength λ = 700, 750, 850, and 900 nm, respectively.

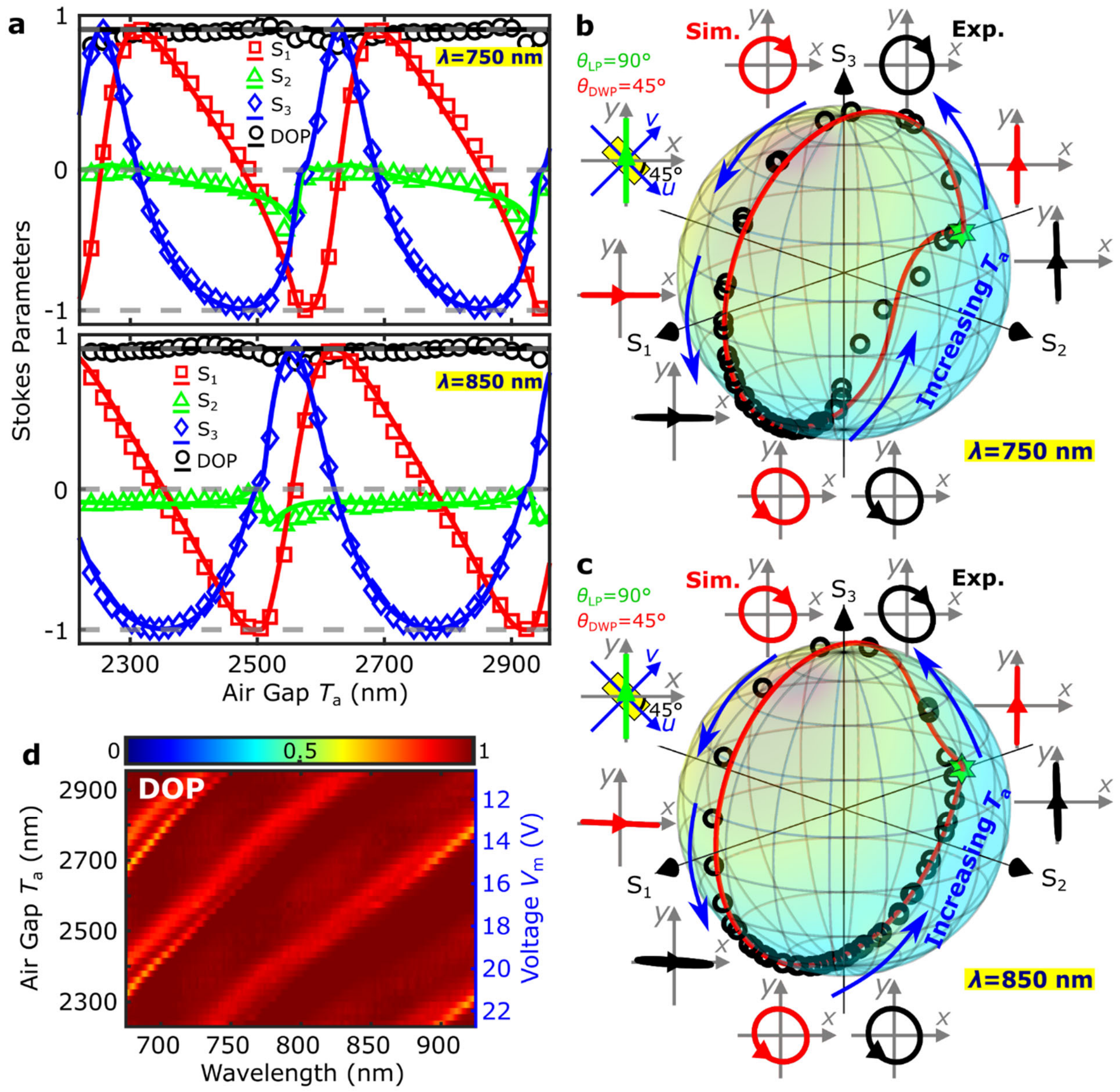

Fig. S10. Polarization transformations for fixed input LP |*y*> state (θ_{LP} = 90°) and DWP orientation θ_{DWP} = 45°. a Calculated (lines) and measured (markers) normalized Stokes parameters (S_1, S_2, S_3) as a function of air gap T_a for light wavelength λ = 750 and 850 nm, respectively. **b-c** Calculated (lines) and measured (circles) polarization trajectories on the Poincaré spheres mapping the reflected light polarization evolution shown in (**a**) for two different light wavelengths of λ = 750 (**b**) and 850 (**c**) nm, respectively. As the air gap T_a increases, the reflected polarization state revolves repeatedly around the sphere. The green stars mark the input LP state: |*y*>. The calculated (red) and measured (black) polarization ellipses illustrate the polarization contrast between the main orthogonal polarization states (|*x*> and |*y*>, |*r*> and |*l*>) realized by tuning the air gap T_a. **d** Measured DOP of the reflected light as a function of the air gap T_a (or actuation voltage V_m) and light wavelength λ.

S6. Higher quality of four typical polarization states (i.e., |*x*>, |*y*>, |*r*>, |*l*>) with DWP orientation θ_{DWP} =46°, for fixed incident LP state |*y*> (θ_{LP} = 90°).

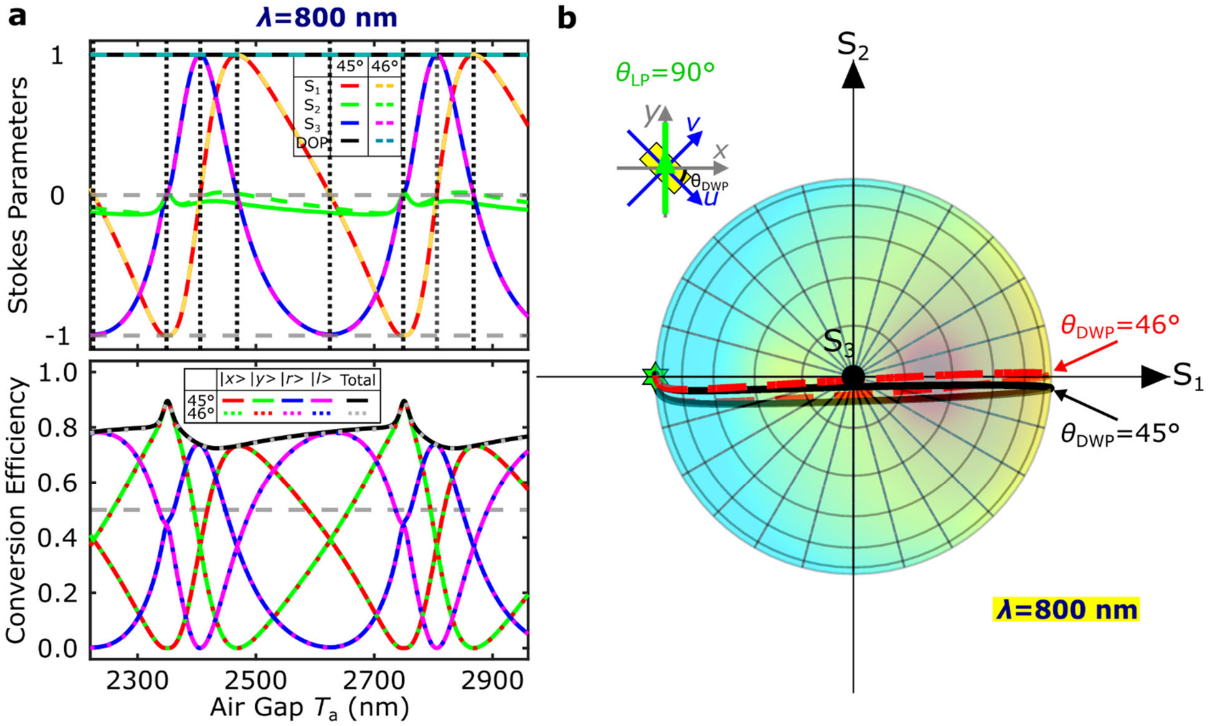

Fig. S11. Polarization transformations for linearly |*y*> polarized light incidence and DWP oriented at respective θ_{DWP}=45° and 46°. a Calculated normalized Stokes parameters (above) and polarization conversion efficiencies (below) as a function of the air gap T_a for the light wavelength λ = 800 nm, with θ_{DWP}=45° and 46°, respectively. **b** Top view of the calculated polarization trajectories on the Poincaré sphere mapping the reflected light polarization evolution shown in (**a**). The green star marks the incident LP state: |*y*>. The solid and dashed lines are corresponding to DWP oriented at θ_{DWP}=45° and 46°, respectively.

Supplementary Table 2. Calculated polarization purity and polarization conversion efficiency of the four typical polarization states (i.e., |*x*>, |*y*>, |*r*>, |*l*>), with DWP oriented at respective θ_{DWP} = 45° and 46°, for linearly |*y*> polarized light incidence.

θ_{DWP}	45°				46°			
	S_1	S_2	S_3	Efficiency	S_1	S_2	S_3	Efficiency
|*x*>	**0.9983**	-0.0577	0.0049	0.7325	**0.9999**	0.0120	0.0049	0.7316
|*y*>	**-1.0000**	0.0072	0.0004	0.8929	**-1.0000**	0.0072	0.0004	0.8931
|*r*>	0.0097	-0.0507	**0.9987**	0.7372	0.0103	-0.0155	**0.9998**	0.7363
|*l*>	0.0060	-0.1249	**-0.9921**	0.7809	0.0091	-0.0901	**-0.9959**	0.7790

S7. Versatile polarization transformations

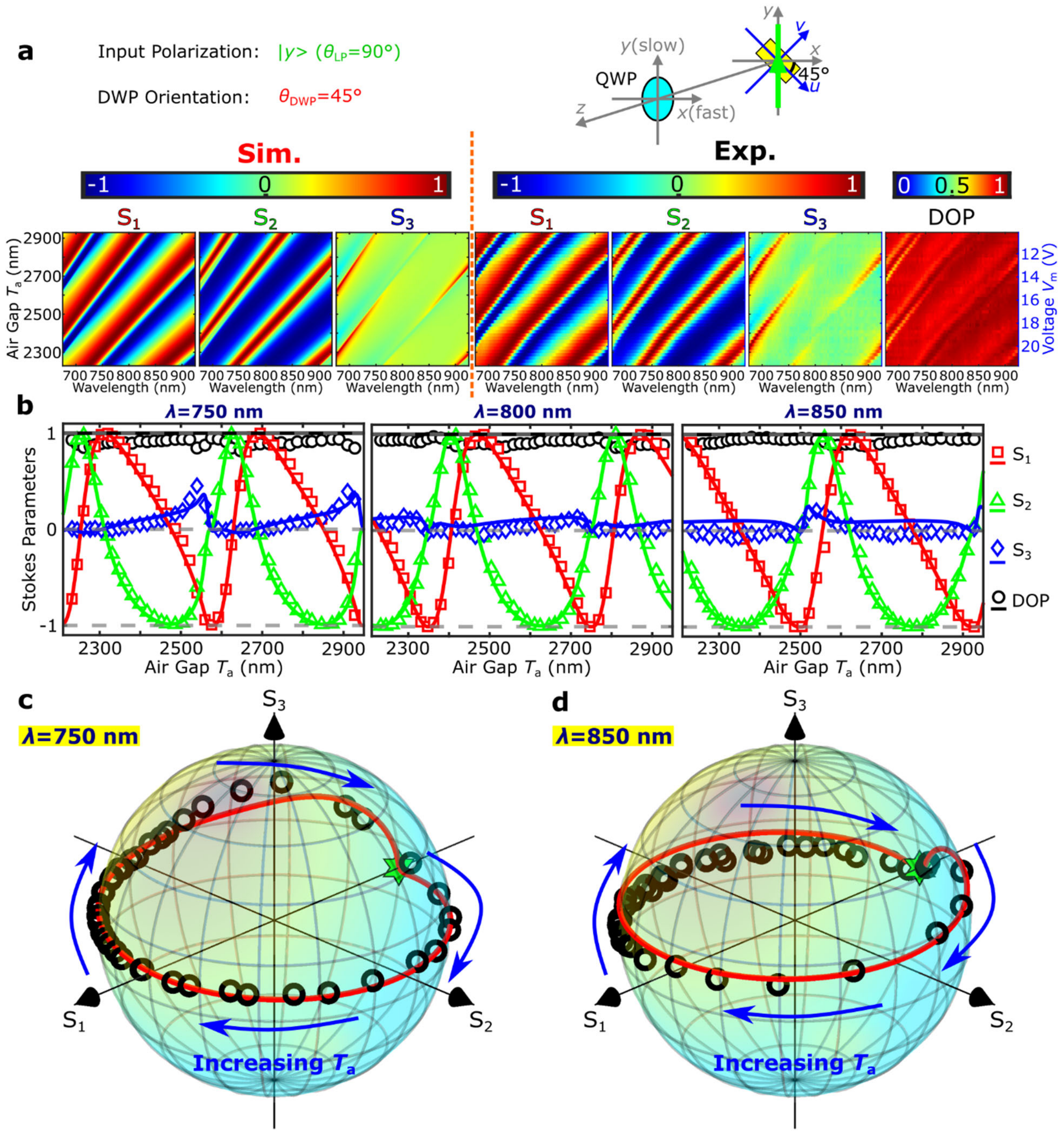

Fig. S12. Continuous linear polarization rotation. a Calculated (left) and measured (right) normalized Stokes parameters (S_1, S_2, S_3) and DOP as a function of the air gap T_a (or actuation voltage V_m) and light wavelength λ. The upper-right inset illustrate the QWP-DWP component: a QWP with fixed QWP orientation (fast axis along x direction) is placed in front of the DWP (θ_{DWP} = 45°), excited by an incident LP state |y>. **b** Calculated (lines) and measured (markers) normalized Stokes parameters as a function of T_a for light wavelengths λ = 750, 800 and 850 nm, respectively. **c-d** Calculated (lines) and measured (circles) polarization trajectories on the Poincaré sphere realized by tuning the air gap T_a for light wavelength λ = 750 (**c**) and 850 (**d**) nm, respectively.

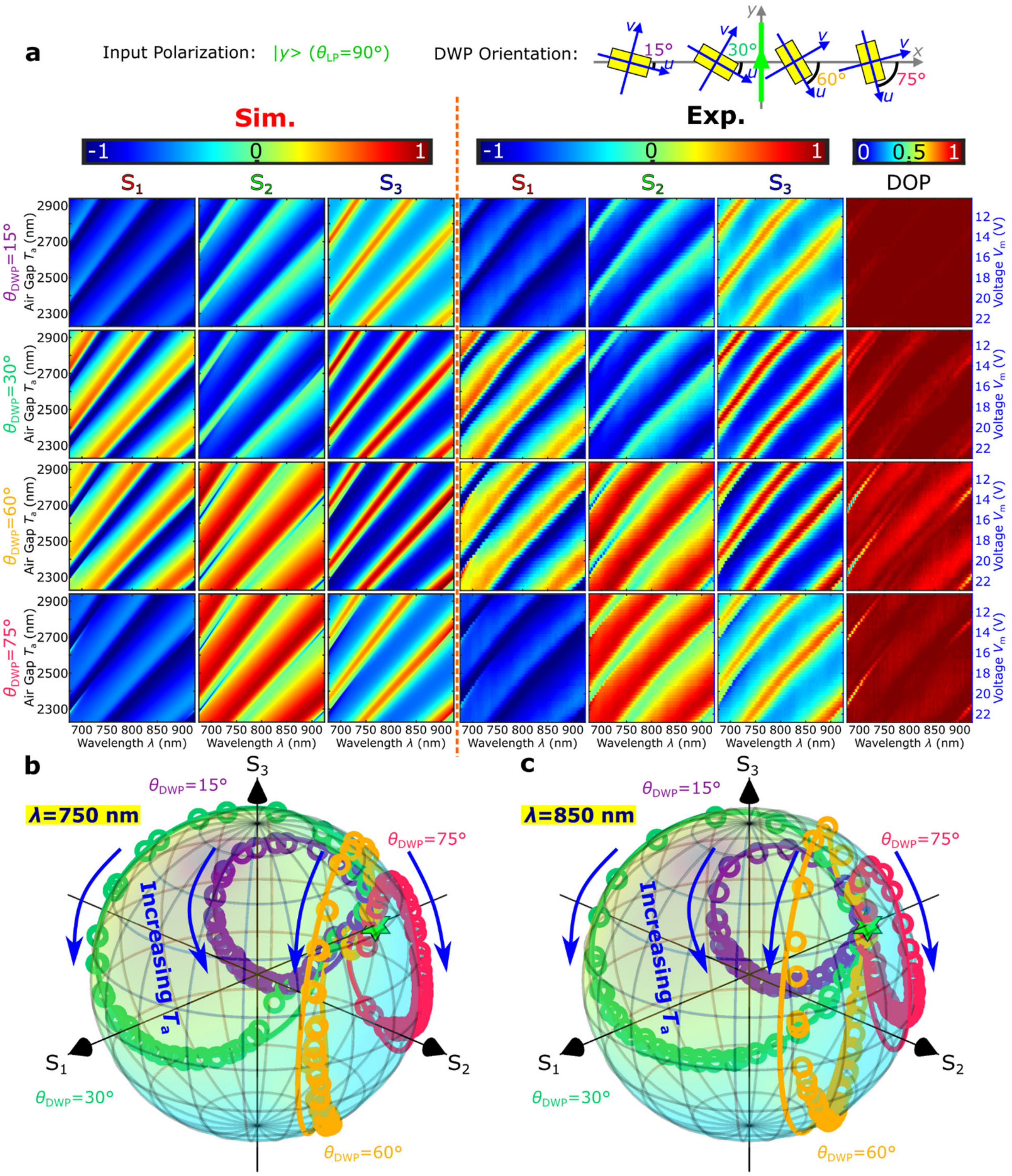

Fig. S13. Versatile polarization transformations for fixed input LP |*y*> state (θ_{LP} = 90°) and different DWP orientations. a Calculated (left) and measured (right) normalized Stokes parameters (S_1, S_2, S_3) and DOP as a function of the air gap T_a (or actuation voltage V_m) and light wavelength λ, for four different DWP orientations of θ_{DWP} = 15°, 30°, 60° and 75°, respectively. **b-c** Calculated (lines) and measured (circles) polarization trajectories on the Poincaré sphere realized by tuning the air gap T_a, with four different DWP orientations θ_{DWP} = 15° (purple), 30° (green), 60° (yellow) and 75° (red), for λ = 750 (**b**) and 850 (**c**) nm, respectively.

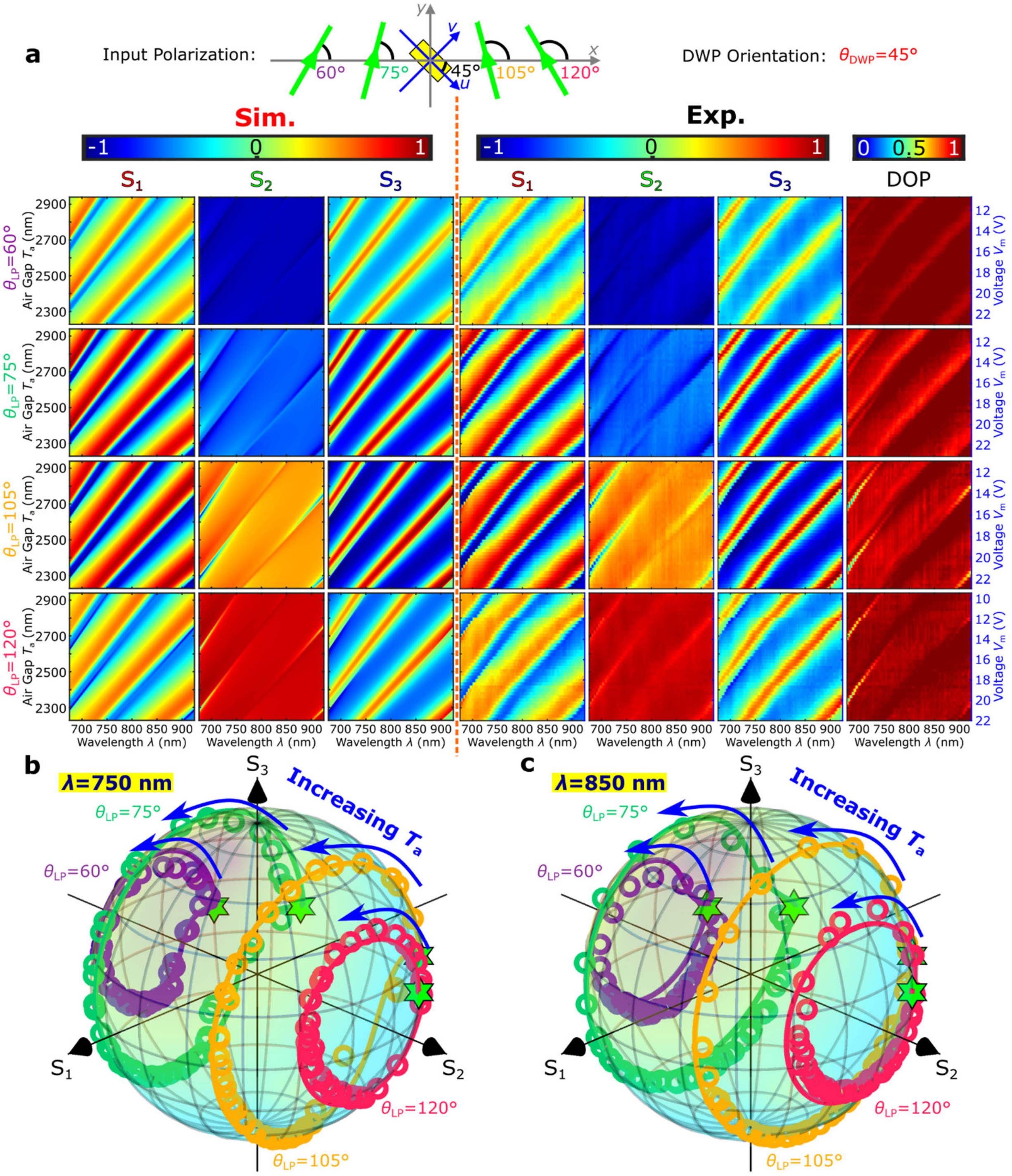

Fig. S14. Versatile polarization transformations for fixed DWP orientation ($\theta_{DWP} = 45°$) and different input LP states. a Calculated (left) and measured (right) normalized Stokes parameters (S_1, S_2, S_3) and DOP as a function of the air gap T_a (or actuation voltage V_m) and light wavelength λ, for four different input LP states of θ_{LP} = 60°, 75°, 105° and 120°, respectively. **b-c** Calculated (lines) and measured (circles) polarization trajectories on the Poincaré sphere realized by tuning the air gap T_a, with four different input LP states θ_{LP} = 60° (purple), 75° (green), 105° (yellow), 120° (red), for λ = 750 (**b**) and 850 (**c**) nm, respectively.

References:

1. Yu, P., Li, J. & Liu, N. Electrically tunable optical metasurfaces for dynamic polarization conversion. *Nano Lett.* **21**, 6690–6695 (2021).

2. Biswas, S., Grajower, M. Y., Watanabe, K., Taniguchi, T. & Atwater, H. A. Broadband electro-optic polarization conversion with atomically thin black phosphorus. *Science* **374**, 448–453 (2021).

3. Nicholls, L. H. *et al.* Ultrafast synthesis and switching of light polarization in nonlinear anisotropic metamaterials. *Nat. Photon.* **11**, 628–633 (2017).

4. Schirato, A. *et al.* Transient optical symmetry breaking for ultrafast broadband dichroism in plasmonic metasurfaces. *Nat. Photon.* **14**, 723–727 (2020).

5. Wang, K. *et al.* High contrast, femtosecond light polarization manipulation in epsilon-near-zero material coupled to a plasmonic nanoantenna array. *ACS Photonics* **8**, 2791–2799 (2021).

6. Zanotto, S. *et al.* Optomechanics of chiral dielectric metasurfaces. *Adv. Opt. Mater.* **8**, 1901507 (2020).

7. Chen, H.-T. Interference theory of metamaterial perfect absorbers. *Opt. Express* **20**, 7165 (2012).

8. Ameling, R. & Giessen, H. Microcavity plasmonics: Strong coupling of photonic cavities and plasmons. *Laser Photon. Rev.* **7**, 141–169 (2013).

9. Berkhout, A. & Koenderink, A. F. Perfect absorption and phase singularities in plasmon antenna array etalons. *ACS Photonics* **6**, 2917–2925 (2019).

10. Thongrattanasiri, S., Koppens, F. H. L. & García De Abajo, F. J. Complete optical absorption in periodically patterned graphene. *Phys. Rev. Lett.* **108**, 047401 (2012).

11. Yao, Y. *et al.* Electrically tunable metasurface perfect absorbers for ultrathin mid-infrared optical modulators. *Nano Lett.* **14**, 6526–6532 (2014).
