# Supplementary material for: Full-range birefringence control with piezoelectric MEMS-based metasurfaces
Source: Nat Commun. 2022 Apr 19;13:2071. doi: 10.1038/s41467-022-29798-0 (PMC9018774; doi:10.1038/s41467-022-29798-0)
Supplement: Supplementary file 3 — Inventory of Supporting Information [file 41467_2022_29798_MOESM3_ESM.pdf]

Inventory of Supporting Information

Full-range birefringence control with piezoelectric MEMS-based metasurfaces

Chao Meng, [1†] Paul C. V. Thrane, [1,2†] Fei Ding,[1*] and Sergey I. Bozhevolnyi[1*]

[1]*Centre for Nano Optics, University of Southern Denmark, Campusvej 55, Odense DK-5230, Denmark*

[2]*SINTEF Microsystems and Nanotechnology, Gaustadalleen 23C, 0737 Oslo, Norway*

[†]These authors contributed equally to this work

[*]Corresponding author emails: feid@mci.sdu.dk (F.D.); seib@mci.sdu.dk (S.I.B.)

1. A supplementary information file;
2. Supplementary Video S1;
3. Supplementary Video S2;
4. Supplementary Video S3;
5. Supplementary Video S4;
6. Supplementary Video S5;
7. Supplementary Video S6;
